# Controlled Synthesis of Triangular Submicron-Sized CeO_2_ and Its Polishing Performance

**DOI:** 10.3390/ma17092001

**Published:** 2024-04-25

**Authors:** Xingzi Wang, Ning Wang, Zhenyu Zhang, Xianmin Tan, Yuanyuan Zheng, Juanyu Yang

**Affiliations:** 1 National Engineering Research Center for Rare Earth, GRIREM Advanced Materials Co., Ltd., Beijing 100088, China; wxz18700427323@163.com (X.W.); ning@grirem.com (N.W.); zhangzhenyu@grirem.com (Z.Z.); tanxianmin@grirem.com (X.T.); zhengyuanyuan@grirem.com (Y.Z.); 2 Rare Earth Functional Materials (Xiong’an) Innovation Center Co., Ltd., Xiong’an, Baoding 071700, China; 3 General Research Institute for Nonferrous Metals, Beijing 100088, China

**Keywords:** CeO_2_, CMP, Ce^3+^ concentration, material removal rate, morphology

## Abstract

CeO_2_ is widely used in the field of chemical–mechanical polishing for integrated circuits. Morphology, particle size, crystallinity, and Ce^3+^ concentration are crucial factors that affect polishing performance. In this study, we successfully synthesized two novel triangular CeO_2_ abrasives with similar particle sizes (600 nm) but different morphologies and Ce^3+^ concentrations using a microwave-assisted hydrothermal method with high-concentration raw materials, and no surfactants or template agents were added. It is generally believed that CeO_2_ with a higher Ce^3+^ concentration leads to better polishing performance. However, the results of polishing indicate that CeO_2_ synthesized at 200 °C, despite its lower Ce^3+^ concentration, demonstrates outstanding polishing performance, achieving a polishing rate of 324 nm/min, and the S_a_ of Si wafers decreased by 3.6% after polishing. This suggests that, under similar particle size conditions, the morphology of CeO_2_ plays a dominant role in the mechanical effects during the polishing process. Additionally, compared to commercial polishing slurries, the synthesized samples demonstrated better polishing performance. This indicates that, in CMP, the pursuit of smaller spherical abrasives may not be necessary. Instead, the appropriate shape and particle size can better balance the material removal rate and surface roughness.

## 1. Introduction

Integrated circuit (IC) manufacturing requires exceptionally flat wafer surfaces. Chemical–mechanical polishing (CMP) is the predominant technology for achieving nanoscale global planarization, widely applied in integrated circuit manufacturing. The key elements of CMP include a polishing slurry, a polishing pad, a polishing machine, and the process parameters [1]. CMP involves both mechanical abrasion of abrasive particles and chemical corrosion in the slurry to remove materials, achieving a highly planar surface by leveraging the advantages of both mechanical and chemical polishing [2]. Therefore, the polishing slurry plays a crucial role in determining the material removal rate (MRR) and surface roughness (S_a_) throughout the CMP process. The selection of abrasive materials is a crucial step in CMP process design, and is extensively explored by researchers [3]. 

Cerium oxide (CeO_2_) is widely used in IC polishing due to its variable valence, high reactivity, slightly lower hardness than SiO_2_, and strong affinity for SiO_2_ [4,5,6]. It can form Ce-O-Si bonds with SiO_2_, accelerating polishing and exhibiting a very high selectivity removal ratio. In current research and the literature, various methods for preparing nanoscale or submicron CeO_2_ are explored, including precipitation [7,8,9], hydrothermal synthesis [10,11,12], sol–gel [13], and microemulsion [14]. Currently, precipitation and hydrothermal methods are prevalent. In the preparation process, lower concentrations (below 0.1 M) and template agents or surfactants are typically used to achieve a specific morphology and a uniform distribution of CeO_2_ [15,16,17].

From the perspective of chemical polishing, current research shows that Ce^3+^ can form Ce-O-Si bonds with the SiO_2_ surface, thereby increasing MRR. Consequently, it is widely acknowledged that CeO_2_ with a higher Ce^3+^ concentration exhibits superior polishing performance [18,19,20,21]. On the mechanical removal front, the morphology and size of abrasives are critical factors determining the removal rate. Therefore, morphology, particle size, and Ce^3+^ concentration are all crucial factors that affect polishing performance [22]. However, in general, CeO_2_ with different morphologies has different Ce^3+^ concentrations [23], leading to a lack of in-depth research in the existing literature on the dominant role of these factors in CMP.

Furthermore, in the current CMP process, there is a common pursuit of smaller-sized spherical CeO_2_ abrasives, as they can reduce scratches and decrease S_a_. However, generally speaking, non-spherical abrasives exhibit higher removal rates compared to spherical abrasives [22]. Therefore, there is a need to seek CeO_2_ with both distinctive morphology and moderate particle size to achieve a balance between MRR and S_a_.

This study proposes a novel CeO_2_ morphology suitable for IC CMP, capable of balancing MRR and S_a_. Notably, during its preparation, there is no need to add surfactants or template agents, and it features a high raw material concentration, facilitating industrial production and reducing manufacturing costs. Additionally, our research investigates the polishing performance of CeO_2_ with different morphologies and Ce^3+^ concentrations but similar particle sizes. The results indicate that mechanical action predominates during the CMP process, and the polishing performance of CeO_2_ is significantly influenced by its morphology and particle size.

## 2. Experimental

### 2.1. Material Preparation

All the reagents were of analytical grade and were used without further purification: cerium carbonate (Ce_2_(CO_3_)_3_·6H_2_O ≥ 99.99%, Hebei Chengfeng Chemical Co., Ltd., Wuhan, China), ammonium bicarbonate (NH_4_HCO_3_ ≥ 99.5%, Xilong Science Co., Ltd., Shantou, China), and nitric acid (HNO_3_, 68 wt.%–70 wt.%, Modern Oriental Fine Chemistry Co., Ltd., Beijing, China). Cerium carbonate was dissolved in HNO_3_ to prepare a 1.0 mol/L cerium carbonate solution, and ammonium bicarbonate was dissolved in deionized water to prepare a 1.8 mol/L solution.

In a typical synthesis procedure, a 1.0 mol/L ammonium bicarbonate solution was added to a 1.8 mol/L cerium nitrate solution at a rate of 16 mL/min while stirring vigorously for 10 min. The mixture was transferred to a 300 mL Teflon-lined autoclave and microwave-assisted hydrothermal treatment was performed at 150 °C and 200 °C for 24 h, respectively, followed by natural cooling to room temperature. The resulting white precipitates were collected by centrifugation, washed 3–4 times with deionized water, and dried at 100 °C in air for 24 h. Finally, the obtained white powder was calcined in air at 600 °C for 2 h in a muffle furnace to obtain the CeO_2_ samples.

### 2.2. Structural Characterization

The morphology and size of the samples were examined using a scanning electron microscope (SEM, S-4800; Hitachi; Kyoto, Japan). The average particle size was determined using a nanoparticle size analyzer (LA-960; Horiba; Kyoto, Japan).

Thermogravimetric and differential thermal analyses were carried out under an air atmosphere from 25 to 1000 °C at a heating rate of 10 °C min^−1^, utilizing a thermal analyzer (TG, TGA/DSC 3+; Mettler Toledo; Columbus, OH, USA). The phase information and lattice structure of the samples were characterized by an X-ray diffractometer with Cu K radiation in the 2θ range of 10–90° (XRD, Smart Lab 9 kW; Japan Science Co., Ltd.; Kyoto, Japan). Additionally, a microscopic confocal Raman spectrometer (Lab RAM Aramis; Horiba Jobin Yvon; Paris, France) was employed, with a 325 nm laser as the excitation source and the analyzed wavenumber range spanning from 200 to 800 cm^−1^.

High-resolution transmission electron microscopy (TEM, Talos F200X; Thermo Fisher Scientific; Waltham, MA, USA) and selected-area electron diffraction (SAED) were utilized to visualize the morphology and lattice information of CeO_2_. These methods offer detailed insights into the internal structure and arrangement of the crystals.

### 2.3. Polishing Test

Two-inch-diameter Si wafers were employed as the workpiece for CMP, utilizing a porous polyurethane pad on the CMP polisher. After polishing, the Si wafers were sequentially cleaned with water, ethanol, and ammonia in an ultrasonic cleaner. Subsequent measurements were conducted after the wafer dried. The specific parameters for the polishing process are detailed in Table 1. The change in mass of the Si wafer before and after CMP was used to indirectly calculate MRR according to Equation (1).

(1) $$\mathrm{MRR}=(\Delta m\;\times \;H)\;/\;m_{0}t$$

where *H* and *m*_0_ represent the height (nm) and the mass of the Si wafer before CMP, respectively; Δm is the weight difference of the Si wafer before and after CMP; and *t* is the polishing time (min). The S_a_ of the Si wafer after polishing was measured by a 3D optical surface profiler (New View™ 9000; Zygo; Middlefield, CT, USA).

Polishing slurries were prepared with a concentration of 1 wt.% using HT-150 and HT-200 as polishing abrasives separately. An amount of 0.5 wt.% of sodium hexametaphosphate (SHMP) was added as a dispersant to each slurry and the pH was adjusted to 6 using HNO_3_ or ammonia solution. Two commercial polishing slurries (CC1 and CC2) were used for polishing under the same parameters for comparison.

## 3. Results and Discussion

### 3.1. Morphology and Structure Characterization of CeO_2_ Abrasives

In this study, we utilized cerium carbonate solution and ammonium bicarbonate as raw materials, and employed a microwave-assisted hydrothermal method. By controlling the hydrothermal synthesis temperatures at 150 °C and 200 °C, respectively, we obtained two carbonate precursors, which were subsequently calcined at 600 °C to obtain the final products.

**Figure 1:**
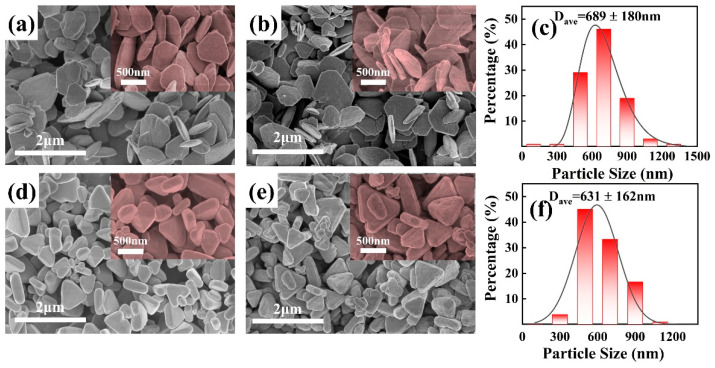
SEM images of HT-p-150 (**a**), HT-150 (**b**), HT-p-200 (**d**), and HT-200 (**e**), and particle size distribution of HT-150 (**c**) and HT-200 (**f**).

The morphological features of the samples subjected to hydrothermal treatment at 150 °C and 200 °C were investigated using SEM, as illustrated in Figure 1.

Figure 1a,c, respectively, showcase the carbonate precursors synthesized under hydrothermal conditions at 150 °C and 200 °C, labeled as HT-p-150 and HT-p-200. Subsequently, Figure 1b,d display the CeO_2_ samples obtained after calcination at 600 °C, designated as HT-150 and HT-200. It can be observed that the samples synthesized at different hydrothermal temperatures all present a nearly monodisperse state, with little to no aggregation. Compared to the precursors, the morphology and size of the products after calcination remain largely unchanged but exhibit more pores/voids on the surface, attributed to the release of water and carbon dioxide throughout the calcination process [24]. 

As shown in Figure 1b,c, HT-150 appears as flake-like truncated triangular shapes with a certain degree of curvature on the surface, and an average height of 689 nm. In contrast, as depicted in Figure 1e,f, HT-200 exhibits triangular shapes with a thickness of approximately 200 nm, featuring a relatively flat surface and rounded angles of the triangles, with an average height of 631 nm. 

**Figure 2:**
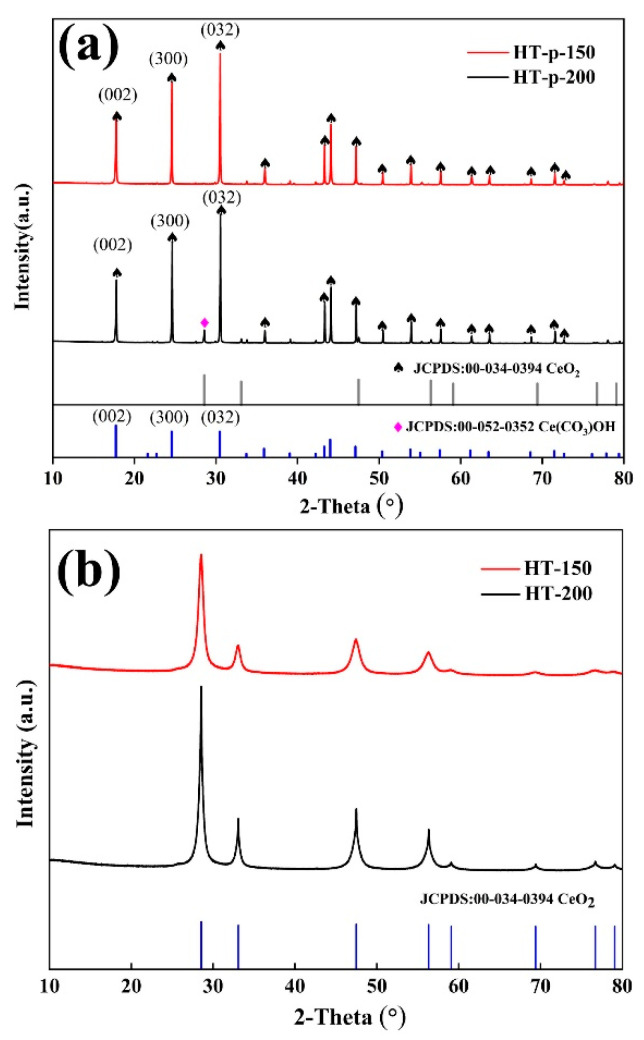
XRD patterns of precursors and calcination products (**a**,**b**).

To determine the chemical composition of the prepared precursors at different hydrothermal temperatures and the calcined products, XRD analysis was conducted as shown in Figure 2.

As shown in Figure 2a, the XRD diffraction pattern of HT-p-150 matches the hexagonal CeCO_3_OH (JCPDS No. 52-0352), and no other phases were detected. In contrast, the XRD pattern of HT-p-200 corresponds to both the hexagonal CeCO_3_OH and the cubic fluorite CeO_2_, indicating partial oxidative decomposition of CeCO_3_OH as the hydrothermal temperature reaches 200 °C. Additionally, as depicted in Table 2, in the precursor samples, the intensity ratios of the (300) and (032) planes to the (002) plane are both higher than the proportions observed in the standard material (JCPDS No. 52-0352), indicating the presence of a preferred orientation in the growth of crystal planes within the synthesized precursor samples [25,26].

Moving to Figure 2b, the XRD pattern of the calcination products, well-crystallized at 600 °C, shows that both HT-150 and HT-200 can be accurately indexed to pure cubic fluorite CeO_2_ (JCPDS No. 034-0394). Moreover, HT-200 demonstrates higher crystallinity compared to HT-150.

**Figure 3:**
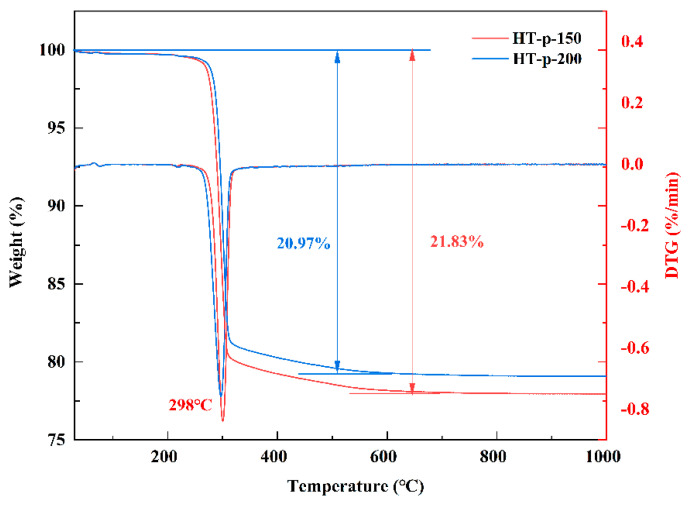
TG−DTG curves of HT-p-150 and HT-p-200.

The thermal stability of the precursors synthesized at 150 °C and 200 °C was investigated by TG analysis, as shown in Figure 3.

It can be observed that the weight losses of precursors HT-p-150 and HT-p-200 are 20.97% and 21.87%, respectively. These weight losses are higher than the theoretical weight loss calculated based on Equation (2) (20.73%) [27], attributed to the presence of adsorbed water and crystalline water in the precursors. Additionally, due to the presence of CeO_2_ in the precursor HT-p-200, its weight loss is relatively lower. A significant weight loss of precursors HT-p-150 and HT-p-200 is observed at 298 °C, believed to be associated with the decomposition of cerium carbonate. 

Beyond 600 °C, the weight of the sample shows minimal further decline, indicating complete decomposition of the precursor. As the calcination temperature increases, the particles of the product are more prone to growth and aggregation. Therefore, based on the TG—DTG curves, it is reasonable that the calcination temperature for precursors was chosen at 600 °C.

(2) $$4CeCO_{3}OH+O_{2}\to 4CeO_{2}+4CO_{2}+2H_{2}O$$

**Figure 4:**
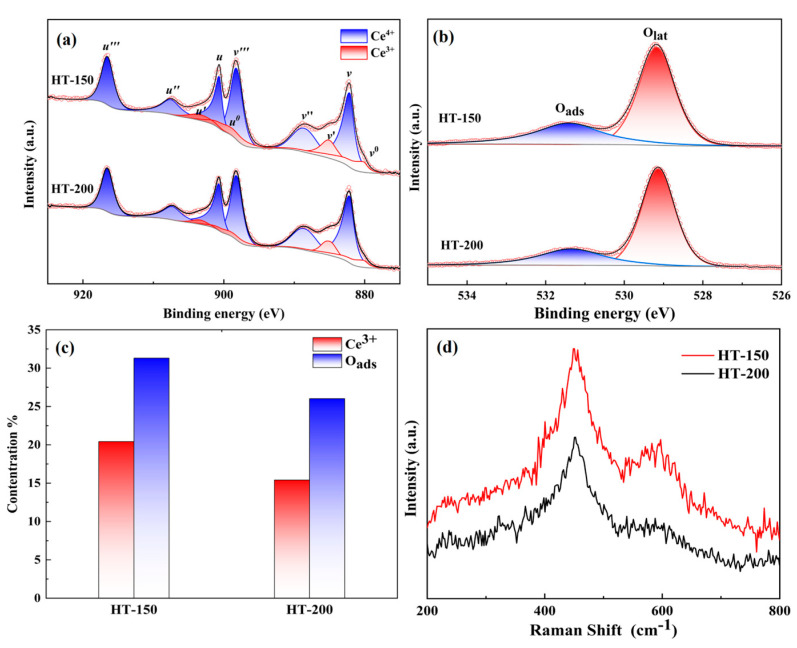
The fitted Ce 3d spectra (**a**) and O1s (**b**), and the statistical results after fitting (**c**) and Raman (**d**).

XPS analysis was carried out to investigate the Ce oxidation state (Ce^3+^/Ce^4+^) at the surface of CeO_2_. For quantitative calculations of the Ce^3+^ concentration in CeO_2_ particles, all the XPS Ce 3d spectra were deconvoluted into ten separate peaks using the mixed Gaussian–Lorentzian function. The fitted spectrum, shown in Figure 4a, designates *v* and *u* for the Ce 3d5/2 and Ce 3d3/2 graphs, respectively. The *v*^0^, *u*^0^, *v*′, and *u*′ peaks are attributed to Ce^3+^, while the remaining peaks correspond to Ce^4+^ ions [28].

The Ce^3+^ concentration can be calculated from semi-quantitative formula by using the area under fitted peaks [29]:

(3) $$\left[{Ce}^{3+}\right]=A\left(v^{0}\right)+A(u^{0})+A\left(v^{\prime }\right)+A(u^{\prime })$$

From Figure 4a,c, the calculated Ce^3+^ concentration values are 20.42% and 15.41% for HT-150 and HT-200, respectively. In other words, the concentration of Ce^3+^ in the CeO_2_ prepared at a hydrothermal temperature of 150 °C is higher than that prepared at a hydrothermal temperature of 200 °C.

We also analyzed the O1s spectrum, and the fitted spectrum is illustrated in Figure 4b. From this, we can clearly observe two types of oxygen peaks around 529.2 eV and 531.4 eV, which correspond to lattice oxygen (Olat) and surface—adsorbed oxygen (Oads) [15,23]. The area and intensity of the Oads peak are relevant to the OVs in the host lattice. The Oads ratio of HT-150 is calculated as 31.31%, which is higher than that of HT-200 (26.02%), suggesting that the O1s result is in line with the results from the Ce 3d spectra.

Figure 4d shows Raman spectra of the synthesized CeO_2_, complementing the structural characterization and valence information. In the test, we used an incident laser wavelength of 325 nm UV light, mainly because UV Raman is more sensitive to defect sites in ceria than visible Raman [30,31]. This is due to the resonance Raman effect, since ceria strongly absorbs light in the UV region [32]. We can see two main peaks centered at 451 and 600 cm^−1^. The peak near 451 cm^−1^ represents the Raman F_2g_ mode, which originates from oxygen stretching vibrations and is strongly affected by the grain size [33], as established by the Grüneisen relation [34]. It could contribute to confinement and inhomogeneous strain effects, which influence the mode position and are responsible for the asymmetrical broadening of the Raman peak [33,35]. The active vibration peak near 600 cm^−1^ in the Raman spectrum is the defect—induced (D) mode, ascribed to the intrinsic oxygen vacancies because of the presence of Ce^3+^. The ratio of ID/IF_2g_ is used to reflect the concentration of oxygen vacancies [36]. Based on the data presented in Figure 4d, the ID/IF_2g_ values for HT-150 and HT-200 were 0.346 and 0.219, respectively. This suggests that the concentration of oxygen vacancies or Ce^3+^ in the sample synthesized at 150 °C is higher than that in the sample synthesized at 200 °C [32].

**Figure 5:**
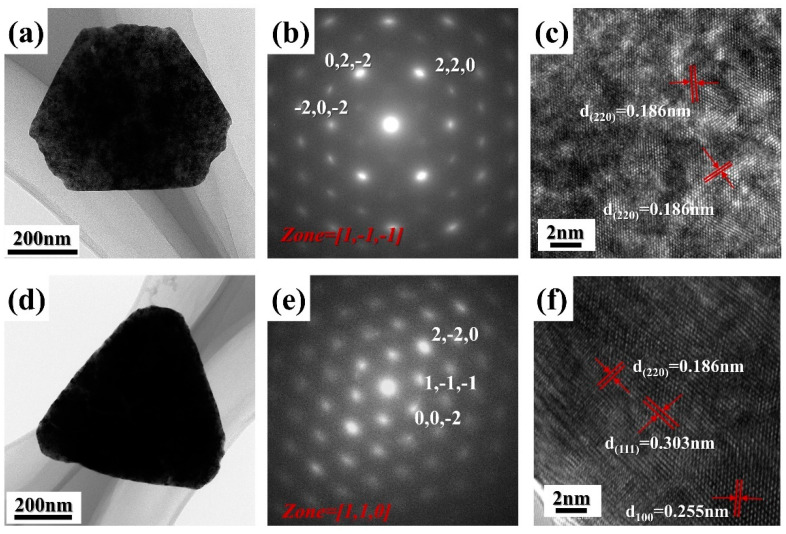
TEM, SEAD, and HRTEM of HT-150 (**a**–**c**), and HT-200 (**d**–**f**).

Figure 5a,d show the bright—field TEM images of CeO_2_ prepared at 150 °C and 200 °C, respectively.

It can be seen that the morphology and size of CeO_2_ observed by TEM are consistent with SEM. Figure 5b,e show the corresponding selected—area electron diffraction (SAED) patterns, revealing that CeO_2_ prepared at different hydrothermal temperatures contains single crystals with an FCC structure. Figure 5c displays the HRTEM image of the sample prepared at 150 °C, indicating lattice spacings of 0.186 nm corresponding to the (110) crystal planes of CeO_2_, consistent with SAED. Figure 5f presents the HRTEM image of CeO_2_ prepared at 200 °C, revealing lattice spacings of 0.255 nm, 0.303 nm, and 0.186 nm corresponding to the (100), (111), and (110) crystal planes of CeO_2_ [22,37]. The DFT calculations indicate that the (111), (110), and (100) planes are the three most thermodynamically stable surfaces of CeO_2_, with a vacancy formation energy sequence of (110) < (100) < (111) [38,39]. This suggests that the (100) and (110) crystal planes have higher defect concentrations [40]. Since the main exposed crystal plane of the sample prepared at 150 °C is the (110) plane, its surface Ce^3+^ concentration is higher than that of the 200 °C sample, which is primarily dominated by the (111), (110), and (100) planes. This is consistent with the results of XPS and Raman analysis.

### 3.2. The Polishing Performance of CeO_2_ Abrasives

MRR and S_a_ are the two most important indicators for evaluating the polishing performance of polishing materials.

**Figure 6:**
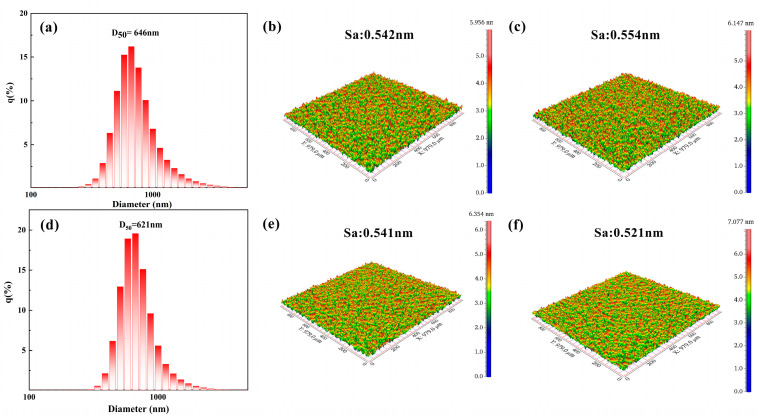
The particle size distribution of polishing slurries HT-150 (**a**) and HT-200 (**d**), and the 3D surface contour map of the Si wafer before polishing (**b,e**), and after polishing with slurry HT-150 (**c**) and slurry HT-200 (**f**).

HT-150 and HT-200 were dispersed in water to prepare a 1 wt.% polishing slurry, with the addition of SHMP as a dispersant. The particle size distributions of the polishing slurry are shown in Figure 6a,d. It can be observed that both polishing slurries exhibit single—peak distributions. The median particle diameters (D_50_) of HT-150 and HT-200 are 646 nm and 621 nm, respectively, with polydispersity indexes of 0.253 and 0.136, indicating that the dispersibility of the synthesized CeO_2_ exhibits excellent dispersion characteristics, especially for HT-200.

By measuring the weight change in the Si wafer during the polishing process, the MRRs for HT-150 and HT-200 were indirectly calculated, resulting in 216 nm/min and 324 nm/min, respectively. It is evident that the MRR of HT-200 is significantly higher than that of HT-150. Subsequently, an analysis of its S_a_ was conducted, and the typical 3D surface contour map and S_a_ of the Si wafer before and after polishing using HT-150 and HT-200 slurries, respectively, are shown in Figure 6b,c,e,f. Before polishing, the S_a_ values of the Si wafer are nearly the same. After polishing with HT-150, the S_a_ of the Si wafer slightly increases to 0.554 nm. When using HT-200 as the abrasive, the S_a_ decreases to 0.521 nm.

These results indicate that CeO_2_ prepared at a hydrothermal temperature of 200 °C exhibits superior polishing performance, striking a balance between a higher material removal rate and a lower S_a_. This contradicts the conventional understanding that a higher Ce^3+^ concentration typically leads to better polishing performance. This suggests that the difference in performance is likely attributed to the influence of morphology, crystallinity, and particle size distribution on mechanical properties during the chemical–mechanical polishing process, outweighing the effects of Ce^3+^ concentration.

**Figure 7:**
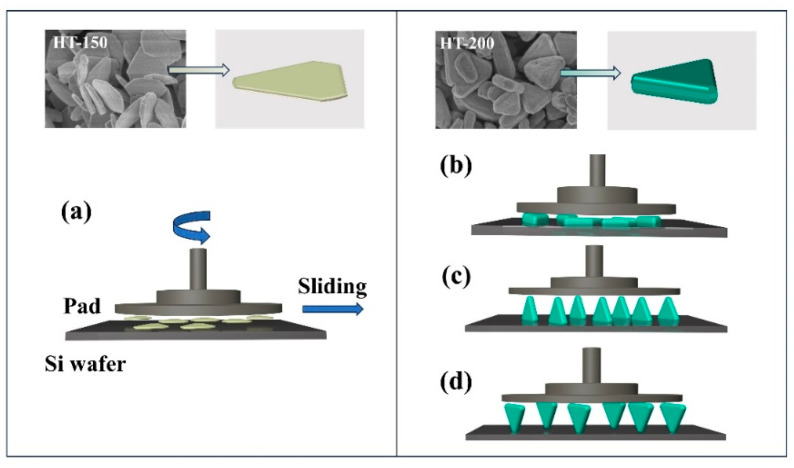
Schematic diagram of abrasive particles in the CMP: HT-150 (**a**) and HT-200 (**b**–**d**).

HT-150 is a flake—like material, reflecting its polishing state during CMP as shown in Figure 7a.

The thinner HT-150 abrasive has a larger contact area with the wafer, which leads to enhanced interfacial adhesion. And it is difficult for the tangential force of the machine to act on the thinner abrasive, which may make part of the abrasives ineffective. This may lead to degraded polishing performance. Additionally, compared to HT-200, HT-150 has a broader particle size distribution with relatively more large particles. This may lead to a higher likelihood of scratches and consequently higher S_a_ after polishing. In contrast, HT-200, with a certain thickness (~200 nm), may exhibit different states during polishing. As illustrated in Figure 7b, the triangular plane comes into contact with the Si wafer, providing superior S_a_ due to its relatively flat and smooth contact surface. In the polishing state shown in Figure 7c, the contact mode between CeO_2_ and the Si wafer surface is linear, increasing the contact pressure and thereby elevating the MRR. Finally, in the state depicted in Figure 7d, the abrasive particles have point contact with the Si wafer, exerting maximum pressure. This significantly enhances the MRR [22,41], while the rounded angles of the triangle prevent excessive scratching. Furthermore, compared to HT-150, HT-200 has a smaller average particle size and a greater percentage of particles smaller than the mean, effectively “filling in the gaps” between the larger particles. Therefore, HT-200 may be more effective in grinding than HT-150. The combined effect of the above polishing states allows HT-200 to have both a higher MRR and a superior S_a_.

**Figure 8:**
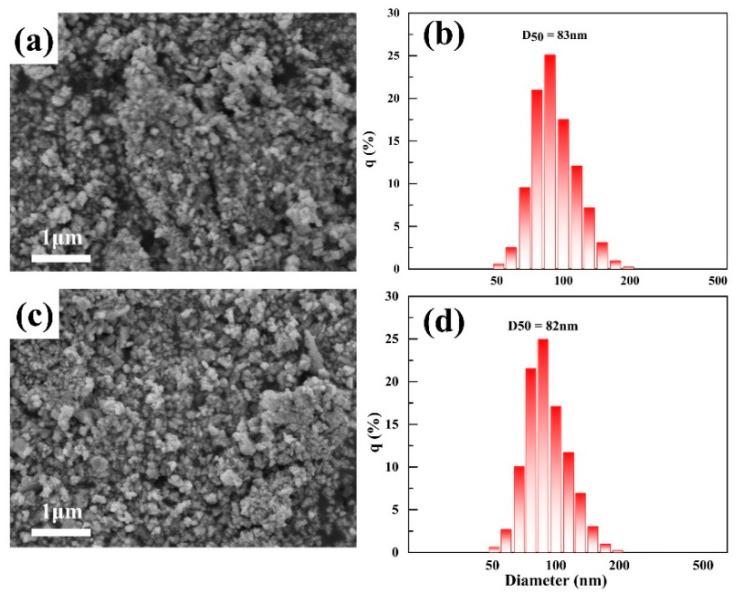
SEM images of commercial polishing abrasives CC1 (**a**) and CC2 (**c**), and the particle size distribution of polishing slurries CC1 (**b**) and CC2 (**d**).

At present, the CeO_2_ slurry commonly used in IC generally consists of small particles. Figure 8 displays two common polishing slurries, which have a spherical particle shape with a median particle diameter (D50) of approximately 80 nm. We selected these two slurries as the control groups and conducted polishing studies under the same polishing process.

The average MRR and S_a_ values are summarized in Table 3 to evaluate the polishing performance quantitively. The MRRs for HT-150, HT200, CC1, and CC2 are 216 nm/min, 324 nm/min, 136 nm/min, and 154 nm/min, respectively, accompanied by S_a_ values of 0.554, 0.521, 0.592, and 0.847 nm. Clearly, the MRR of HT-200 is 210% higher compared to CC2, and S_a_ is reduced by 11.9% compared to CC1, demonstrating optimal polishing performance.

This implies that HT-200 is capable of effectively balancing MRR and surface roughness, S_a_.

In comparison, the MRR and S_a_ of the commercial polishing slurries in the control group are both inferior to those of the CeO_2_ slurries synthesized in this study. This may be attributed to their predominantly spherical morphology and smaller particle sizes [42]. The polishing mechanism of spherical CeO_2_ involves rolling friction, resulting in a relatively lower MRR. Moreover, smaller particle sizes lead to an increased specific surface area and enhanced agglomeration, thereby causing challenges in subsequent cleaning and leaving a higher residue of CeO_2_ particles on the surface of Si wafers [42].

In summary, the CeO_2_ prepared in this study demonstrates favorable polishing performance, particularly with HT-200. This attests to the superiority of the morphological characteristics of CeO_2_ represented by HT-200 in polishing applications.

## 4. Conclusions

This study successfully synthesized two novel types of nearly monodisperse CeO_2_ at a high concentration of raw material without the addition of any dispersants. Both particle sizes were around 600 nm. At 150 °C, both types of CeO_2_ exhibited flake—like truncated triangular shapes with a Ce^3+^ concentration of 20.42%. At 200 °C, they took on a triangular form with a thickness of approximately 200 nm and a Ce^3+^ concentration of 15.41%. Polishing performance characterization revealed MRRs of 214 nm/min and 324 nm/min for CeO_2_ synthesized at 150 °C and 200 °C, respectively. Surprisingly, CeO_2_ with a higher Ce^3+^ concentration did not exhibit superior polishing performance as expected. Therefore, we speculate that factors such as morphology and crystallinity play a dominant role in the mechanical effects during CMP.

Furthermore, there is no need to actively pursue CeO_2_ with a smaller spherical shape. Polishing slurries prepared with smaller abrasives have noticeable drawbacks, such as increased surface area and enhanced agglomeration, leading to challenges in cleaning and a higher level of residue. Although spherical abrasives can reduce scratches during polishing, their disadvantage is apparent as the MRR is not as high as that of non—spherical abrasives. Therefore, in CMP, selecting CeO_2_ with a specific morphology and appropriate particle size can balance the MRR and S_a_ more appropriately. It is evident that the triangular CeO_2_ prepared at 200 °C, as presented in this paper, is a favorable choice.

## Figures and Tables

**Table 1:** Polishing parameters.

Parameter	Specifications
Head rotating speed	45 rpm
Pad rotating speed	15 rpm
Slurry flow rate	50 mL/min
Polishing pressure	5.98 Psi
Polishing time	2 min

**Table 2:** XRD diffraction peak intensity ratio of precursors.

	HT-p-150	HT-p-200	Standard (JCPDS No. 52-0352)
Intensity ratio of (300)/(002)	1.50	1.79	0.79
Intensity ratio of (032)/(002)	1.85	2.35	0.79

**Table 3:** MRR and S_a_ data of various slurries.

	HT-150	HT-200	CC1	CC2
MRR (nm/min)	216	324	136	154
S_a_ (nm)	0.554	0.521	0.592	0.847

## Data Availability

Data are contained within the article.
